# Household physical activity and cancer risk: a systematic review and dose-response meta-analysis of epidemiological studies

**DOI:** 10.1038/srep14901

**Published:** 2015-10-07

**Authors:** Yun Shi, Tingting Li, Ying Wang, Lingling Zhou, Qin Qin, Jieyun Yin, Sheng Wei, Li Liu, Shaofa Nie

**Affiliations:** 1 Department of Epidemiology and Biostatistics, and the Ministry of Education Key Lab of Environment and Health, School of Public Health, Tongji Medical College, Huazhong University of Science and Technology, Wuhan, Hubei, China

## Abstract

Controversial results of the association between household physical activity and cancer risk were reported among previous epidemiological studies. We conducted a meta-analysis to investigate the relationship of household physical activity and cancer risk quantitatively, especially in dose-response manner. PubMed, Embase, Web of science and the Cochrane Library were searched for cohort or case-control studies that examined the association between household physical activity and cancer risks. Random–effect models were conducted to estimate the summary relative risks (RRs), nonlinear or linear dose–response meta-analyses were performed to estimate the trend from the correlated log RR estimates across levels of household physical activity quantitatively. Totally, 30 studies including 41 comparisons met the inclusion criteria. Total cancer risks were reduced 16% among the people with highest household physical activity compared to those with lowest household physical activity (RR = 0.84, 95% CI = 0.76–0.93). The dose-response analyses indicated an inverse linear association between household physical activity and cancer risk. The relative risk was 0.98 (95% CI = 0.97–1.00) for per additional 10 MET-hours/week and it was 0.99 (95% CI = 0.98–0.99) for per 1 hour/week increase. These findings provide quantitative data supporting household physical activity is associated with decreased cancer risk in dose-response effect.

Cancer has been the leading cause of disease burden worldwide, and the increase rates of morbidity and mortality are faster than before in global population. It is estimated that 12.7 million cancer cases and 7.6 million cancer deaths occurred in 2008^1^. Around 90% of cancers have been related to environmental exposures and lifestyle. Physical activity is one of the important known lifestyle-related factors. The World Health Organization (WHO) states that, compared to less active adults, more active individuals have lower rates of all-cause mortality, coronary heart disease, high blood pressure, stroke, type 2 diabetes, metabolic syndrome, colon and breast cancer, and depression. Accordingly, the WHO recommends at least 2.5 h of moderate intensity, 1.25 h of vigorous intensity or an equivalent combination of moderate and vigorous intensity aerobic physical activity throughout the week to reduce the risk of chronic non-communicable diseases (NCDs)^2^. Household physical activity might make a larger contribution to total physical activity, especially among women^3^. It is important whether household physical activity could affect health benefits. In recent years, there is growing evidence suggesting an association between household physical activity and cancer risk^4^,^5^,^6^,^7^,^8^,^9^,^10^,^11^,^12^,^13^,^14^,^15^,^16^,^17^,^18^,^19^,^20^,^21^,^22^,^23^,^24^,^25^,^26^,^27^,^28^,^29^,^30^,^31^,^32^,^33^. Nevertheless, epidemiological evidence on the relationship has not been systematically assessed. Moreover, many of the individual studies have grouped participants into quantitatively designated categories of household physical activity based on energy expenditure^6^,^9^,^18^,^25^,^26^,^28^,^29^,^31^,^33^ or time expenditure^5^,^7^,^8^,^10^,^13^,^15^,^16^,^17^,^18^,^22^,^24^, making it possible to quantify the association between household physical activity and cancer risk in a dose-dependent manner.

Therefore, we conducted a meta-analysis of observation comparisons assessing the association between household physical activity and cancer risk quantitatively to provide more detailed and useful evidence for public health guidelines.

## Results

### Study Selection

**Figure 1:**
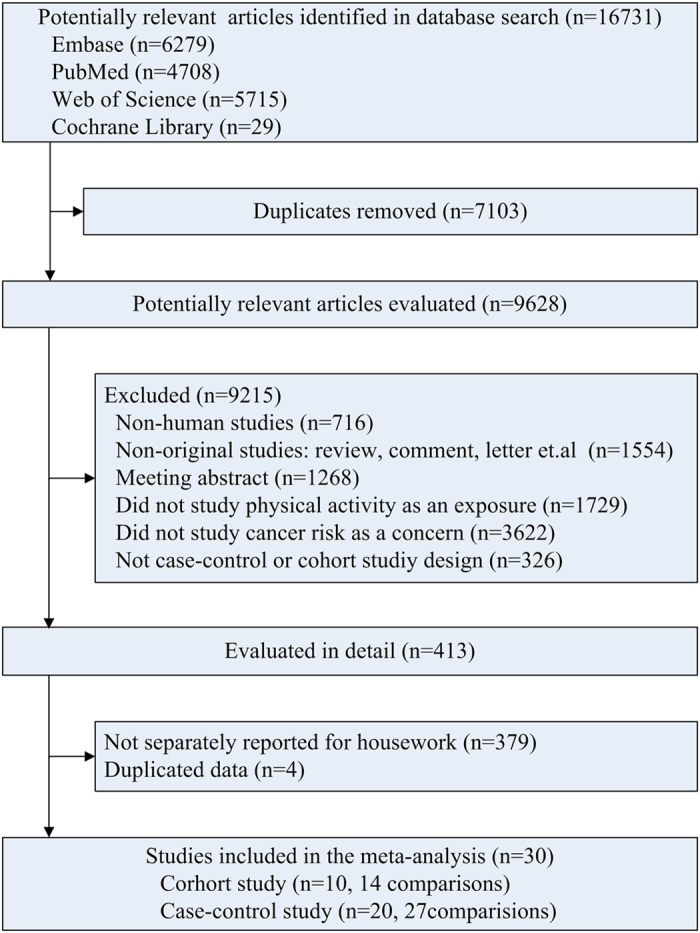
Flowchart of study selection.

Figure 1 shows study selection process and results from the literature search. We identified 16,731 potentially relevant articles by the search strategy from the four databases. After exclusion of papers that did not meet the inclusion criteria based on information included in the abstracts, we obtained 413 full articles of potentially relevant studies. After full text evaluation, 4 studies were excluded due to duplicated data. 379 studies were further excluded because they reported total physical activity or other physical activity subgroup but not separately reported for household physical activity. Finally, 30 studies^4^,^5^,^6^,^7^,^8^,^9^,^10^,^11^,^12^,^13^,^14^,^15^,^16^,^17^,^18^,^19^,^20^,^21^,^22^,^23^,^24^,^25^,^26^,^27^,^28^,^29^,^30^,^31^,^32^,^33^ were included in the primary meta-analysis.

### Study Characteristics

General characteristics of the 30 included studies^4^,^5^,^6^,^7^,^8^,^9^,^10^,^11^,^12^,^13^,^14^,^15^,^16^,^17^,^18^,^19^,^20^,^21^,^22^,^23^,^24^,^25^,^26^,^27^,^28^,^29^,^30^,^31^,^32^,^33^ which totaled 41 comparisons had been shown in Supplementary Table S1. Briefly, 14 comparisons^14^,^15^,^16^,^17^,^19^,^24^,^26^,^27^,^28^,^29^ were cohort study design, and 27 comparisons^4^,^5^,^6^,^7^,^8^,^9^,^10^,^11^,^12^,^13^,^18^,^20^,^21^,^22^,^23^,^25^,^30^,^31^,^32^,^33^ were case-control study design. 7 comparisons^5^,^11^,^14^,^16^,^21^,^27^,^32^ presented the estimates for males, 29 comparisons^4^,^5^,^6^,^7^,^8^,^9^,^10^,^13^,^14^,^15^,^17^,^18^,^19^,^20^,^22^,^26^,^27^,^28^,^29^,^30^,^31^,^32^,^33^ for females. 27 comparisons^4^,^5^,^8^,^9^,^12^,^14^,^15^,^16^,^17^,^18^,^19^,^20^,^21^,^24^,^25^,^27^,^28^,^29^,^32^ were constructed in Europe, 7 comparisons^7^,^13^,^22^,^23^,^26^ in Asia, 6 comparisons^6^,^11^,^30^,^31^ in America and 1 comparisons^33^ in Africa. Among those comparisons on relationship between household physical activity and cancer risk, breast cancer was estimated mostly, which included 21 comparisons^6^,^7^,^8^,^9^,^10^,^15^,^18^,^20^,^22^,^26^,^28^,^29^,^30^,^31^,^33^, and endometrial cancer was estimated in 4 comparisons^4^,^13^,^17^,^19^. In addition, colorectal cancer^5^,^16^, lung cancer^14^,^25^, lymphoid neoplasma^12^,^27^, pancreatic cancer^32^, prostate cancer^11^,^21^, gastric cancer^23^,^24^, and esophageal carcinoma^24^ were estimated in the rest comparisons. 20 and 16 comparisons provided quantitative estimates of household physical activity categories in the form of energy expenditure (MET-hour/week)^6^,^9^,^18^,^25^,^26^,^28^,^29^,^31^,^33^ and time expenditure (hour/week)^5^,^7^,^8^,^10^,^13^,^15^,^16^,^17^,^18^,^22^,^24^, respectively. Results of study quality assessment yielded an average score of 7 for all studies. The proportions of low, moderate, and high quality were 0.0% (0/30), 30% (9/30), and 70% (21/30).

### Highest versus lowest analysis

**Figure 2:**
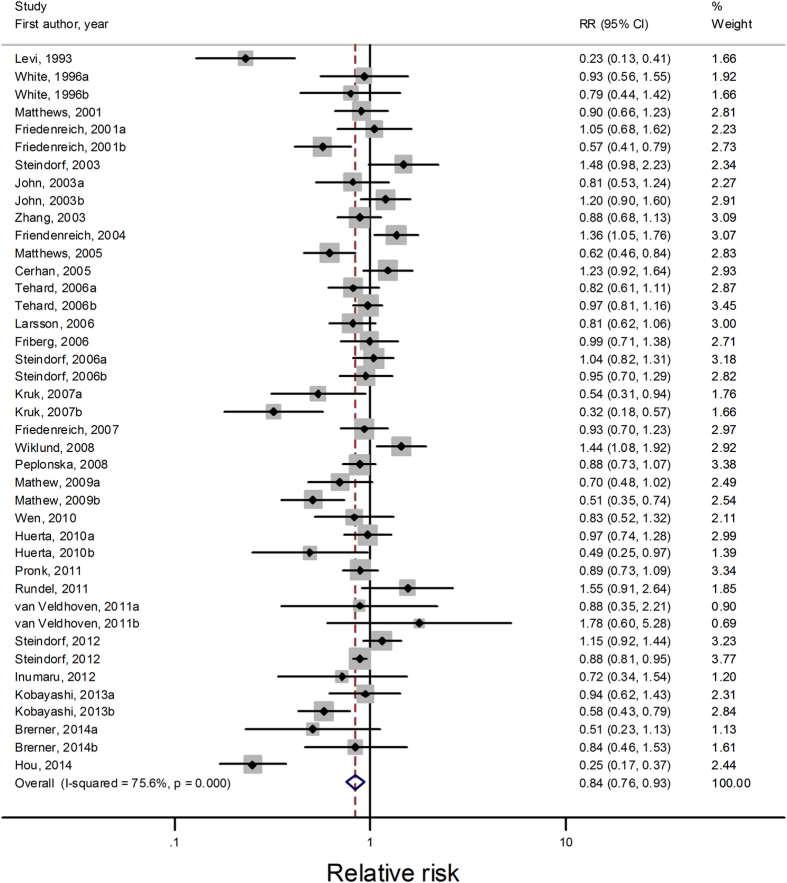
Forest plots of highest versus lowest meta-analysis on the relationship between household physical activity and cancer risk.

All studies with 41 comparisons^4^,^5^,^6^,^7^,^8^,^9^,^10^,^11^,^12^,^13^,^14^,^15^,^16^,^17^,^18^,^19^,^20^,^21^,^22^,^23^,^24^,^25^,^26^,^27^,^28^,^29^,^30^,^31^,^32^,^33^ were included for highest versus lowest analysis, which consisted of 2,242,789 participants and 33,949 cancer cases. Compared with lowest household physical activity level, the highest level had a summary RR of 0.84 (95% CI = 0.76–0.93, *I*^*2*^_for heterogeneity_ = 75.6%; Fig. 2). Table 1 presents the results of subgroup analyses. A significant inverse association between household physical activity and cancer risk was found in both cohort studies and case-control studies. However, pooled estimate from cohort comparisons was more conservative with the summary relative risks of 0.92 (95% CI = 0.87–0.97, *I*^*2*^_for heterogeneity_ = 0.1%; Table 1), compared with 0.77 (95% CI = 0.65–0.92, *I*^*2*^_for heterogeneity_ = 82.4%; Table 1) from case-control studies. Geographically, active household physical activity resulted in cancer risk reduction in Asia (RR = 0.76, 95% CI = 0.65–0.90), but not in America (RR = 0.83, 95% CI = 0.59–1.18) or Europe (RR = 0.92, 95% CI = 0.82–1.02). A significant inverse association between household physical activity and cancer risk was observed in women (RR = 0.78, 95% CI = 0.69–0.88) but not in men (RR = 1.04, 95% CI = 0.84–1.30). When further stratified sex by study design and location, the results also showed significant association for women but non-significant association for men. Besides, we examined whether obesity mediated the inverse relation of household physical activity with cancer risk in subgroup analyses. The inverse association of household physical activity to cancer risk was statistically significant in studies adjusting for BMI/weight (RR = 0.80, 95% CI = 0.71–0.90) but it was not significant in studies without adjustment for BMI/weight (RR = 0.93, 95% CI = 0.78–1.10).

**Figure 3:**
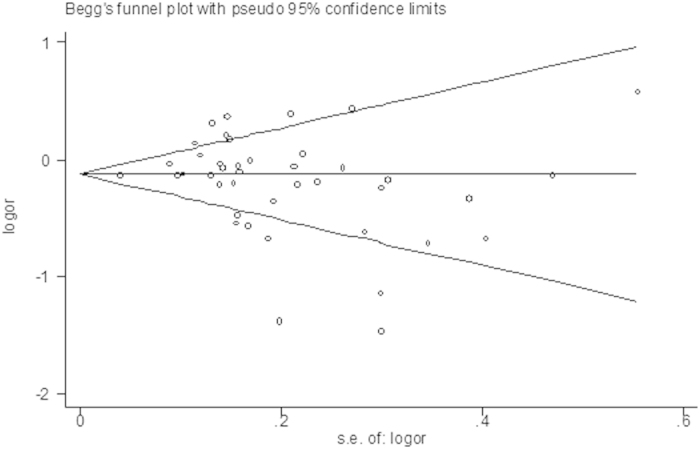
Funnel plots of highest versus lowest meta-analysis on the relationship between household physical activity and cancer risk.

Sensitivity analysis found that the summary RR was not substantially influenced by any of the individual studies when recalculating the overall results by omitting one study each time, with a range from 0.82 (95% CI = 0.75–0.91) to 0.87 (95% CI = 0.80–0.95). Marginal publication bias was indicated by Begg’s test (*P* = 0.052, Fig. 3) but not Egger’s tests (*P* = 0.173).

### Dose-response analysis

#### By MET-hour/week

**Figure 4:**
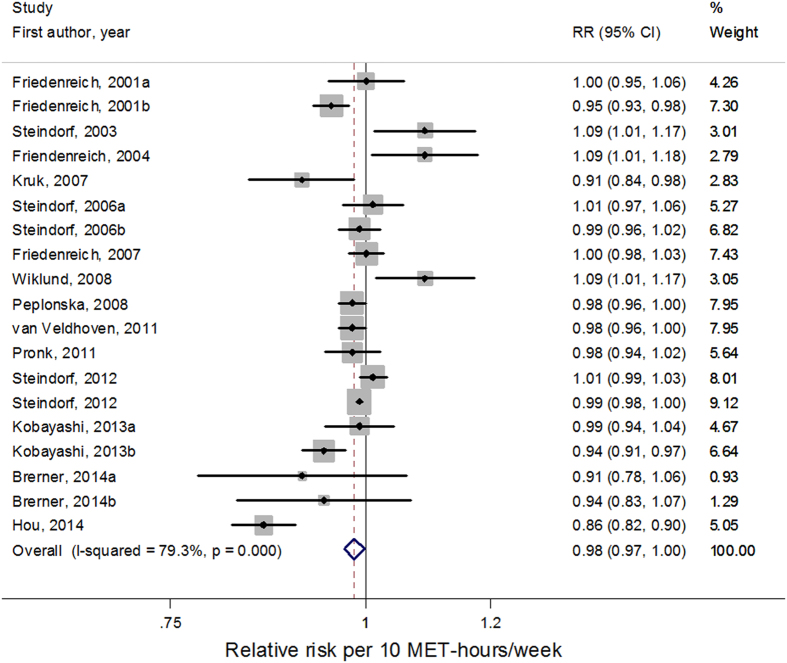
Forest plots of linear dose–response meta-analysis by MET-hour/week on the relationship between household physical activity and cancer risk.

Among the 20 comparisons^6^,^9^,^18^,^25^,^26^,^28^,^29^,^31^,^33^ estimating household physical activity categories quantitatively in the form of energy expenditure, a total of 19 comparisons^6^,^9^,^18^,^26^,^28^,^29^,^31^,^33^ were included in the dose-response analysis by MET-hour/week. One study^25^ was excluded due to lack of category-specific number of cases and person-year/non-cases. Restricted cubic splines model indicated linear association between household physical activity and cancer risk (*P*_for non-linearity_ = 0.89). In the linear dose-response meta-analysis, the summary relative risk for each 10 MET-hours/week increase in household physical activity was 0.98 (95% CI = 0.97–1.00, *I*^*2*^_for heterogeneity_ = 79.3%; Fig. 4). The summary relative risk of a 10 MET-hours/week increment of household physical activity was 0.99 (95% CI = 0.99–1.00, *I*^*2*^_for heterogeneity_ = 5.1%) for cohort studies and 0.98 (95% CI = 0.94–1.01, *I*^*2*^_for heterogeneity_ = 83.3%) for case-control studies, respectively. The pooled relative risk of a 10 MET-hours/week increment of household physical activity was 1.03 (95% CI = 0.97–1.09, *I*^*2*^_for heterogeneity_ = 58.5%) for men and 0.98 (95% CI = 0.96–0.99, *I*^*2*^_for heterogeneity_ = 82.7%) for women, respectively. By study location, the relative risk of a 10 MET-hours/week increment of household physical activity was 0.99 (95% CI = 0.98–1.01, *I*^*2*^_for heterogeneity_ = 58.9%) and 0.98 (95% CI = 0.95–1.02, *I*^*2*^_for heterogeneity_ = 74.9%) for cancer in Europe and America, respectively.

Sensitivity analysis found the summary relative risk was not substantially influenced by any of the individual studies when omitting one study each time. Publication bias was not indicated statistically both with Begg’s test (*P* = 0.972) and Egger’s tests (*P* = 0.577).

#### By hour/week

**Figure 5:**
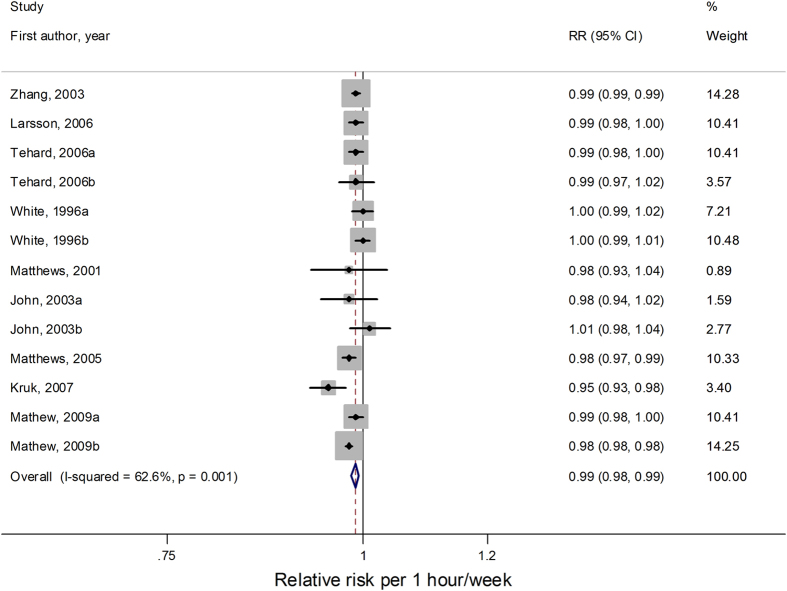
Forest plots of linear dose–response meta-analysis by hour/week on the relationship between household physical activity and cancer risk.

Among the 16 comparisons^5^,^7^,^8^,^10^,^13^,^15^,^16^,^17^,^18^,^22^,^24^ estimating quantitatively household physical activity categories in the form of time expenditure, a total of 13 comparisons^5^,^7^,^8^,^10^,^13^,^15^,^16^,^18^,^22^ were included in the dose-response analysis by hour/week. One comparison^17^ was excluded because its quantitative measures were less than 3 levels. Other two comparisons from Huerta *et al.*’ study^24^ which didn’t provide cut-off points for categories were also excluded. From the pooled results of 13 comparisons, we detected a linear association between household physical activity and cancer risk (*P*_for non-linearity_ = 0.41). In the linear dose-response meta-analysis, the summary relative risk for each 1 hour/week increase in household physical activity was 0.99 (95% CI = 0.98–0.99, *I*^*2*^_for heterogeneity_ = 62.6%; Fig. 5). The summary relative risk of 1 hour/week increment was 0.99 (95% CI = 0.98–1.00,) for both cohort studies and case-control studies. Stratifying by geographic region, the relative risk of 1 hour/week increment was 0.98 (95% CI = 0.97–1.00, *I*^*2*^_for heterogeneity_ = 66.4%) for Europe, 1.00 (95% CI = 0.99–1.01, *I*^*2*^_for heterogeneity_ = 0.0%) for America and 0.99 (95% CI = 0.98–0.99, *I*^*2*^_for heterogeneity_ = 58.8%) for Asia, respectively.

Sensitivity analysis found the summary relative risk was not substantially influenced by any of the individual studies when omitting one study each time and recalculated the overall results. Publication bias was not indicated statistically both with Begg’s test (*P* = 0.393) and Egger’s tests (*P* = 0.761).

## Discussion

To our knowledge, this meta-analysis is the first study to summarize the inverse association between household physical activity and cancer risk. A 16% lower overall cancer risk was detected by comparing the most active with the least active household physical activity. The dose-response meta-analyses revealed an inverse linear relationship between household physical activity and cancer risk. Increment of household physical activity by every 10 MET-hour/week or 1 hour/week was associated with a 1% reduction of cancer risk.

The previous reviews have indicated the important role of physical activity in cancer prevention^34^. And the relationships between physical activity and risk of some types of cancer have been revealed in dose-response manners. A meta-analysis of observational studies by Keum *et al.*^35^ found an increase in leisure-time physical activity by 3 MET-hours/week was associated with a 2% reduced risk of endometrial cancer and an increase by an hour/week was associated with a 5% reduced risk of endometrial cancer. Wu *et al.*’s meta-analysis^36^ revealed that the risk of breast cancer decreased by 2% for every 25 MET-hour/week increment in non-occupational activity, and 3% for every 10 MET-hour/week increment in recreational activity, respectively. Besides, dose-response meta-analyses were performed to detect the association of non-occupational physical activity with ovarian cancer^37^, and moderate to vigorous physical activity with gastroesophageal cancer^38^. Although previous meta-analyses indicated the potential relationship of various domains of activity and cancer risk, few had focused on the association between household physical activity and cancer risk in a dose-response manner. This meta-analysis first indicated the significantly decreased risk of cancer consistently along with the increase of energy expenditure and time expenditure of household physical activity.

Furthermore, we explored the potential inconsistency among different subgroups and revealed some meaningful phenomenons. The inverse association between household physical activity and cancer risk appeared to be more pronounced in case-control studies than cohort studies for binary meta-analysis. However, no obvious gap between the two study designs was found in the linear dose-response analyses. In general, case-control studies are more susceptible to recall and selection bias. And population-based case-control studies are generally less prone to selection bias than hospital-based case-control studies. As we removed hospital-based case-control studies from subgroup analyses by study design, we found the relationship between household physical activity and cancer risk for case-control studies was significantly weakened in highest versus lowest analysis, but was little changed in both of the linear dose-response analyses. The bias caused by hospital-based studies could be an important source for the different results between case-control studies and cohort studies in binary analysis. In addition, we noticed that the inverse association between household physical activity and cancer risk was only found in women but not in men. And further subgroup analyses by study design and location for it suggested the result was robust. Apart from chance, one possible explanation for the finding is the difference in life style between males and females. The proportion of household physical activity accounted for total moderate to vigorous physical activity was much higher among women than that among men^39^. Besides, sex hormone, a mediator between physical activity and cancer risk, may be another possible explanation for the gender difference. Furthermore, the relatively insufficient studies conducted in men may obstacle the detection of the linkage. The inverse association between household physical activity and cancer risk was modestly enhanced when the analysis was restricted to comparisons those were adjusted for BMI/weight. Accumulating evidence suggested that obesity may increase risk of a variety of cancers^40^. It has been estimated that about 20% of all cancers were caused by excess weight^41^. The inverse relationship between household physical activity and cancer risk may be attenuated by the positive relationship between obesity and cancer when studies without adjustment for obesity included.

A number of plausible mechanisms have been proposed to support the cancer prevention role of household physical activity. Hyperinsulinemia and insulin resistance have been associated with increased risk of cancer^42^,^43^. Hyperglycemia indirectly influences cancer cells through upregulation of insulin/IGF-1 and inflammatory cytokines in circulation. However, physical activity could reduce insulin resistance and lower fasting insulin levels, thus inhibited cell proliferation and cellular transformations^43^,^44^. Another explanation is that physical activity could decreases the concentration of inflammatory adipocytokines and increases anti-inflammatory adipocytokines levels alone or by avoidance of adiposity^45^,^46^. And that lowers production of inflammatory markers have been linked with decreased cancer risk^47^. In addition, physical activity might enhance innate and acquired immune response, increase number and activity of macrophages, natural killer and lymphokine-activated killer cells and cytokines, and promote tumor surveillance^48^,^49^. It is also suggested that physical activity could play a role in reducing cancer risk through regulating sex hormones, which have been associated with alterations in cancer risk, especially in breast, endometrial, ovarian and prostate cancer^35^,^37^,^50^.

Several limitations of our study should be acknowledged. Firstly, moderate to high heterogeneity was observed in the overall analysis, which may limit our understanding of the association in various settings and restricts the generalisability of our findings. Therefore, the results should be interpreted with caution. It should be noticed that significant heterogeneity only existed in case-control studies but not in cohort studies, which implied that study design was an important source of the heterogeneity. In addition, subgroup analyses showed that the number of case, measure units of household physical activity and study quality could also bring heterogeneity. Secondly, we failed evaluate household physical activity levels in each study using uniform and accurate measurement due to the heterogeneity in measurement and reporting of physical activity from different studies, which might result in biased results. Thirdly, as this meta-analysis was based on observational studies, although the adjusted estimates were used to pool the results, because they were not fully adjusted, the potential effect from residual confounding could not be ruled out. A primary source of potential residual confounding is likely to stem from confounding variables which were either unmeasured or insufficiently measured in the individual comparisons themselves. Finally, physical activity was assessed by self-report in most included studies, thus misclassification of activity levels is probable and quantitative characterizations should therefore be considered approximate in nature.

Our meta-analysis has several strengths as well. To the best of our knowledge, this is the first meta-analysis that identified the inverse dose–response relationship between household physical activity and cancer risk, and quantized the association within a homogeneous measure of physical activity. We chose to quantify physical activity in units of metabolic equivalent-hours per week and hours per week as they were more frequently reported in studies, which makes the results easier to understand and more conductive. By conducting dose–response analyses in two different measures, intensity of household physical activity itself was shown related to confer a benefit on cancer risk.

In conclusions, the present meta-analysis suggests that household physical activity is associated with a decreased risk of cancer. Approximately, every 10 MET-hours/week or 1 hour/week increase is associated with a 1% reduction in cancer risk. However, caution is needed in interpreting the finding from our meta-analysis because of the inevitable heterogeneity. Further well-designed studies are warranted to confirm the results.

## Methods

### Literature Search

The meta-analysis was performed following the Meta-analysis of Observational Studies in Epidemiology (MOOSE) guidelines^51^. PubMed, Embase, Web of Science and the Cochrane Library were searched for eligible studies published up to June 18, 2015. Search terms including “cancer”, “carcinoma” or “neoplasm” combined with “physical activity”, “exercise”, “household chores” or “housework” and with “risk”, “incidence” or “mortality” were applied in the literature search. No restrictions were imposed on language of publication. References of any related studies and reviews were further screened for potential missing studies.

### Selection criteria

Studies were included if they met the following criteria: (1) was an original study; (2) had a cohort or case-control study design; (3) participants were healthy people at baseline for cohort studies and the outcome was cancer, while in case-control studies, the participants were the patients with primary cancer in cases groups and were healthy people without personal history of cancer in the control group. (4) studied household physical activity as an exposure and cancer risk as an concern; (5) provided the estimate of relative risk (RR) and its 95% confidence interval (CI) or sufficiently data to drive these numbers. Studies were excluded if they were: (1) a case report, review or meeting abstract; (2) cohorts of patients with basic chronic disease (for example, cardiovascular disease). For the dose-response analysis, a quantitative measure of household physical activity for at least three levels, the level-specific number of cases and the level-specific number of either person-years or non-cases had to be provided. Containment relationship in separate publications would be filtered carefully to pick up the one with largest sample size. Two authors (SY and LT) independently read the full text of all articles to determine whether the study met the eligibility criteria outlined above. Disagreements were resolved by discussion.

### Data Extraction

Data were extracted by one author (SY) using a data extraction form and entered into a database. A second author (LT) independently checked these data, and all disagreements were resolved by discussion. For each study, we extracted the effect estimate (reported as a RR or odds ratio [OR]) and its associated 95% CI for the association of household physical activity with cancer risk. If available, we extracted the risk estimates with the greatest adjustment. If a study reported the effect of physical activity at multiple periods or ages and over the lifetime, we used the lifetime result. If a study reported results for males and females separately, both risk estimates were included in the primary analysis. For all comparisons, we abstracted OR/RR and its 95% CI for comparison between the most active group and the least active group. The effect size and 95% CI were inverted for comparisons in which the most active group was used as the reference group. For studies reported household physical activity in the unit of MET-hour/week or hour/week, the quantitative measure range of household physical activity, effect size, 95% CI, the number of cases and person-years or non-cases were abstracted for each activity group. Other extracted data included the first author’s name, the publication year, the category of cancer, the study design (eg. case-control or cohort), the sex of the participants and the location in which the study took place.

### Quality assessment

Two reviewers (SY and LT) completed the quality assessment independently by using Newcastle-Ottawa Scale^52^. This scale awards a maximum of nine points to each study: selection of the study groups (maximum 4 points), comparability of the study populations (maximum 2 points) and ascertainment of the outcome of interest (maximum 3 points). For each point, 1 score indicates higher quality, whereas, 0 representatives lower quality. We assigned scores of 0–3, 4–6 and 7–9 for low, moderate and high quality of studies, respectively.

### Statistical Analysis

Two (highest vs. lowest, dose-response) types of meta-analyses were performed. We combined the case-control and cohort comparisons in the primary meta-analysis because ORs and RRs provide similar estimates of risk when the outcome is rare^53^. The RR was used as a measure of the association between household physical activity and cancer risk. The highest versus lowest analysis was conducted using random-effect model^54^.

Dose-response meta-analyses were performed to estimate the trend from the correlated log RR estimates across levels of household physical activity. First we examined a potential nonlinear association between household physical activity and cancer risk, using restricted cubic splines with four knots at the 5th, 35th, 65th and 95th percentiles of the levels of household physical activity^55^. A *P* value for nonlinearity was calculated by testing the null hypothesis that the coefficient of the second spline was equal to zero. If a liner relationship was found, a summary risk was derived for a 10 MET-hours/week and 1 hour/week increment in household physical activity respectively. The method used to estimate the study-specific RRs was described by Greenland and Longnecker^56^. Then the study-specific risk increments were combined in random-effect meta-analysis. Forest plots of the linear dose–response results were presented for RRs for per 10 MET-hours/week increment and for per 1 hour/week increment. Only comparisons with three or more quantitative exposure levels and using MET-hour/week or hour/week to describe household physical activity dose were included in these analyses. For each study, the median or mean level in each category of household physical activity was assigned to the corresponding relative risk. We assigned the mid-point of the upper and lower boundaries in each category if median or mean were not reported. For studies reported open upper boundaries or uppest boundaries closed with extreme value, we set the half width of this category was the same with nearest category and used the sum of half width and lower boundary as mid-point.

Heterogeneity between comparisons was assessed by Q statistics (*P* < 0.10)^57^ and *I*^2^ was used to quantify the proportion of the total variation due to the heterogeneity^58^. To identify the sources of heterogeneity and explore the potential effects of specific study characteristics on association between household physical activity and cancer risk, subgroup analyses were conducted according to a priori selected variables. Sensitivity analysis was also performed to explore the potential influence of individual study on overall results by omitting one study each time and recalculated the combined RR. Publication bias was assessed by visual inspection of funnel plots as well as statistically with the use of the Begg’s test^59^ and the Egger’s test^60^ (significant level *P* < 0.10). Except where otherwise specified, statistical tests were two-sided and a *P*-value less than 0.05 was considered statistically significant. All analyses were conducted with Stata software (version 12.0; StataCorp, College Station, TX).

## Additional Information

**How to cite this article**: Shi, Y. *et al.* Household physical activity and cancer risk: a systematic review and dose-response meta-analysis of epidemiological studies. *Sci. Rep.* **5**, 14901; doi: 10.1038/srep14901 (2015).

## Supplementary Material

Supplementary Table S1

## Figures and Tables

**Table 1:** Summary results from subgroup analyses for the relationship between highest versus lowest categories of household physical activity and cancer risk.

Subgroups	No. of comparisons	Relative Risk (95% CI)	*P* for heterogeneity	*I*^2^(%)	Begg’s test, Egger’s test
All studies	41	0.84 (0.76–0.93)	<0.001	75.6	0.052, 0.173
Sex					
Male	7	1.04 (0.84–1.30)	0.019	60.6	0.764, 0.344
Female	29	0.78 (0.69–0.88)	<0.001	77.1	0.058, 0.093
Study location					
Europe	27	0.92 (0.82–1.02)	<0.001	67.0	0.182, 0.614
Asia	7	0.76 (0.65–0.90)	0.089	45.4	0.230, 0.205
America	6	0.83 (0.59–1.18)	<0.001	80.6	1.000, 0.767
Study design					
Cohort study	14	0.92 (0.87–0.97)	0.447	0.1	1.000, 0.550
Case-control Study	27	0.77 (0.65–0.92)	<0.001	82.4	0.055, 0.094
Mean age					
≥50	27	0.85 (0.75–0.96)	<0.001	74.3	0.182, 0.332
<50	7	0.81 (0.59–1.11)	<0.001	87.4	1.000, 0.535
No. of cases					
≥500	26	0.89 (0.82–0.98)	<0.001	63.1	0.402, 0.735
<500	15	0.72 (0.54–0.95)	<0.001	84.8	0.060, 0.161
PA measures					
MET-h/wk	20	0.90 (0.77–1.04)	<0.001	80.2	0.928, 0.938
h/wk	16	0.80 (0.71–0.92)	0.002	57.9	0.053, 0.022
No quantitive	4	0.66 (0.32–1.34)	<0.001	88.3	0.308, 0.271
Cancer type					
Breast Cancer	21	0.78 (0.69–0.89)	<0.001	79.1	0.05, 0.125
Endometrial cancer	4	0.64 (0.40–1.03)	<0.001	86.4	0.308, 0.154
Study quality					
score ≥ 8	14	0.91 (0.81–1.03)	0.015	50.6	0.324, 0.556
score < 8	27	0.80 (0.69–0.92)	<0.001	80.8	0.037, 0.202
Adjustment for BMI/Weight					
Yes	27	0.80 (0.71–0.90)	<0.001	80.3	0.002, 0.057
No	14	0.93 (0.78–1.10)	0.002	59.7	0.827, 0.474
