# Supplementary material for: Household physical activity and cancer risk: a systematic review and dose-response meta-analysis of epidemiological studies
Source: Sci Rep. 2015 Oct 7;5:14901. doi: 10.1038/srep14901 (PMC4595663; doi:10.1038/srep14901)
Supplement: Supplementary Table S1 [file srep14901-s1.doc]

**Supplementary Information**

**Household physical activity and cancer risk: a systematic review and dose-response meta-analysis of epidemiological studies**

Yun Shi, Tingting Li, Ying Wang, Lingling Zhou, Qin Qin, Jieyun Yin, Sheng Wei, Li Liu*, Shaofa Nie*

Department of Epidemiology and Biostatistics, and the Ministry of Education Key Lab of Environment and Health, School of Public Health, Tongji Medical College, Huazhong University of Science and Technology, Wuhan, Hubei, China;

* Corresponding author:

Li Liu, Department of Epidemiology and Biostatistics, and the Ministry of Education Key Lab of Environment and Health, School of Public Health, Tongji Medical College, Huazhong University of Science and Technology, No.13 of Hangkong Road, Wuhan, 430030, China.

Tel: +86 2783 693763 ; Fax : +86 2783 693763 ; E-mail: gracefulliuly@163.com

Shaofa Nie, Department of Epidemiology and Biostatistics, and the Ministry of Education Key Lab of Environment and Health, School of Public Health, Tongji Medical College, Huazhong University of Science and Technology, No.13 of Hangkong Road, Wuhan, 430030, China.

Tel: +86 2783 693763; Fax: +86 2783 693763; E-mail: sf_nie@mails.tjmu.edu.cn

Supplementary Table S1 Main characteristics of studies included in the meta-analyses on household physical activity and cancer risk

| **First author, year** | **Study location** | **Sex** | | | **Case-control studies: control type, cohort studies: study name** | **Study size, no. of cases** | **Reference time of PA measurement (cohort studies: follow-up years)** | **Cancer type** | | **PA measures** | **Household physical activity category and relative risk(95% CI)** | **Study quality** | **Covariates in adjusted model** |
| --- | --- | --- | --- | --- | --- | --- | --- | --- | --- | --- | --- | --- | --- |
| **Cohort studies** | | | | | | | | | | | | | |
| Tehard, 2006 | Europe | Female | | | the E3N cohort study | 90,509, 3,424 | Baseline (11.4 years) | Breast cancer | Hours/week | | Light: 0, 1.00 (reference); 1-4, 1.02 (0.82-1.28); 5-13, 0.95 (0.75-1.20); ≥14, 0.82 (0.61-1.11) Heavy: 0, 1.00 (reference); 1-2,0.98 (0.89-1.07); 3-4, 0.94 (0.84-1.06); ≥5, 0.97 (0.81-1.15) | 9 | BMI, menopausal status, HRT use, age at menarche, age at first full-term pregnancy, parity, marital status, use of oral contraceptives, first-degree family history of breast, personal history of benign breast disease, and employed (yes/no) |
| Larsson, 2006 | Europe | Male | | | The cohort of Swedish men(COSM) | 45,906, 496 | Baseline (7.1 years) | Colorectal cancer | Hours/day | | <1, 1.00 (reference); 1-2, 0.90 (0.73-1.10); ≥3, 0.81 (0.62-1.67) | 7 | Age at baseline, education, family history of colorectal cancer , history of diabetes, smoking, aspirin use, BMI and other types of activity |
| Friberg, 2006 | Europe | Female | | | the Swedish Mammography Cohort | 33,723,  199 | Baseline (7.25 years) | Endometrial cancer | Hours/day | | <5, 1.00 (reference); ≥5, 0.99 (0.71-1.38) | 7 | Age in months, parity, history of diabetes, total fruit and vegetable, education, BMI and other types of activity |
| Steindorf, 2006 | Europe | Both | | | EPIC | 416,277, 1083 | Baseline (6.3 years) | Lung cancer | MET-hours/week | | Men: 0-<11.0, 1.0 (reference); 11.0-<23.8, 0.77 (0.60-1.01); 23.8-<43.6, 0.86 (0.67-1.10); ≥43.6, 1.04 (0.82-1.31) Women: 0-<26.0, 1.0 (reference); 26.0-<49.3, 1.04 (0.81-1.33); 49.3-<86.3, 0.90 (0.68-1.18); ≥86.3, 0.95 (0.70-1.30) | 9 | Age, centre, smoking, weight, height, education, total energy intake without energy from alcohol, alcohol intake, intake of fruits, intake of vegetables, intake of red and processed meat and occupational exposure to lung carcinogens, and other types of activity |
| Friedenreich, 2007 | Europe | Female | | | EPIC | 253,023, 689 | Baseline (6.6 years) | Endometrial cancer | MET-hours/week | | <25.12, 1.00 (reference); ≥25.12-<48.08, 0.94 (0.76-1.16); ≥48.08-<85.10, 1.00 (0.80-1.25); ≥85.10, 0.93 (0.70-1.22) | 8 | Age, centre, BMI, age at menarche, menopausal status, age at menopause, number of full-term pregnancies, age at birth of last child, ever use of oral contraceptives, ever use of hormone replacement therapy, education, smoking status, hypertension, diabetes, fruit and vegetable intake, fibre intake, carbohydrate intake, energy intake, and other types of activity |
| Huerta, 2010 | Europe | Both | | | EPIC | 420,449, 80(OAC),  410(GAC) | Baseline (8.8 years) | Oesophageal adenocarcinoma, gastric adenocarcinoma | Hours/week (sex-speciﬁc tertiles) | | Gastric adenocarcinoma: 0, 1 (reference); T1, 1.15 (0.88-1.51); T2, 0.89 (0.66-1.20); T3, 0.97 (0.74-1.28) Oesophageal adenocarcinoma: 0, 1 (reference); T1, 1.04 (0.58-1.88); T2, 0.72 (0.38-1.36); T3, 0.49 (0.25-0.97) | 8 | Age, centre, sex, height, weight, educational level, smoking status, alcohol consumption and daily intake of total energy, fruit, red meat and processed meat. |
| Pronk, 2011 | Asia | Female | | | The Shanghai Women’s Health Study (SWHS) | 73,049,  717 | Baseline (9 years) | Breast cancer | MET-hours/week | | 0-<28.0, 1.0 (reference); 28.0-<42.0, 0.88 (0.72-1.08); 42.0+, 0.89 (0.73,1.09) | 9 | Age, education, family history of breast cancer, age at first birth, and number of pregnancies |
| van Veldhoven, 2011 | Europe | Female | | | EPIC | 343,756, 778 | Baseline (5 years) | Lymphoid neoplasms | MET-hours/week | | Men: <21.02, 1.00 (reference); ≥21.02-<43.6, 0.92 (0.49-1.73); ≥43.6-<80.1, 1.02 (0.41-2.56);≥80.1, 0.88 (0.35-2.21) Women: <21.02, 1.00 (reference); ≥21.02-<43.6, 2.11 (0.73-6.06);≥43.6-<80.1, 1.65 (0.60-4.55); ≥80.1, 1.78 (0.60-5.27) | 8 | Hypertension, hyperlipidaemia, education and diabetes |
| Steindorf, 2012 | Europe | Female | | | EPIC | 345,158, 1059 | Baseline (11.7 years) | Breast Cancer | MET-hours/week | | 26.0, 1.00 (reference); >26.0-49.3, 1.13 (0.96-1.34); >49.3-86.6, 1.14 (0.95-1.38); >86.6, 1.15 (0.92-1.44) | 8 | BMI, age at first period, age at first full term pregnancy, number of full term pregnancies, breast feeding, ever OC pill, menopausal status, age at menopause, use of hormone replacement therapy, alcohol consumption, smoking status, level of school attained, and other types of activity |
| Steindorf, 2012 | Europe | Female | | | EPIC | 257,805, 8034 | Baseline (11.6 years) | Breast cancer | MET-hours/week | | 24.6, 1.00 (reference); >24.6-47.2, 0.98 (0.93-1.05); >47.2-84.0, 0.92 (0.86-0.98); >84.0, 0.88 (0.81-0.95) | 7 | BMI, age at first period, age at first full term pregnancy, number of full term pregnancies, breast feeding, ever OC pill, menopausal status, age at menopause, use of hormone replacement therapy, alcohol consumption, smoking status, level of school attained, and other types of activity |
| **Case-control studies** | | | | | | | | | | | | | |
| Levi, 1993 | Europe | | Female | Hospital-based | | 846,  274 | 10 years before diagnosis/interview | Endometrial cancer | | Subjective level | High, 1 (reference); moderately high, 1.7 (1.0-2.7); moderately low, 2.0 (1.1-3.3); low, 4.2 (2.4-7.5) | 5 | Study centre, age, education, parity, menopausal status, oral contraceptive and oestrogen replacement treatment use, BMI and estimated calorie intake |
| White, 1996 | America | | Both | Population-based | | 881,  444 | Past 10 years | Colorectal cancer | | Hours/week | Men: ≤6 hours/week, 1.00 (reference); >6-12.4, 1.14 (0.68-1.88); >12.4-20, 1.04 (0.63-1.71); >20, 0.93 (0.56-1.56) Women: ≤20, 1.00 (reference); >20-30, 0.89 (0.50-1.60); >30-48, 1.56 (0.92-2.65); >48, 0.79 (0.44-1.42) | 6 | Age |
| Matthews, 2001 | Asia | | Female | Population-based | | 3015,  1459 | Past 10 years | Breast cancer | | Hours/week | 0-1, 1.00 (reference); 2, 1.06 (0.82-1.38); 3, 0.96 (0.74-1.24); 4, 1.03 (0.79-1.34); ≥5, 0.90 (0.66-1.23) | 6 | Age, education, household income, first-degree family history of breast cancer, history of breast fibroadenoma, age at menarche, age at first live birth, and age at menopause. |
| Friedenreich, 2001 | America | | Female | Population-based | | 2470,  1233 | Lifetime | Breast cancer | | MET-hours/week | Premenopausal: 0-<25.6, 1.00 (reference); 25.6-<41.4, 1.11 (0.75-1.63); 41.4-<62.0, 0.83 (0.54-1.26); ≥62.0, 1.05 (0.68-1.62) Postmenopausal: 0-<50.0, 1.00 (reference); 50.0-<71.0, 0.74 (0.55-0.99); 71.0-<99.3, 0.66 (0.49-0.89); ≥99.3, 0.57 (0.41-0.79) | 7 | Age, waist-hip ratio, educational level, ever use of hormone replacement therapy, ever diagnosed with benign breast disease, first-degree family history of breast cancer, ever alcohol consumption, current cigarette smoker, and the other types of activity |
| Steindorf, 2003 | Europe | | Femlae | Population-based | | 1242,  359 | Age 12-30 years | Breast cancer | | MET-hours/week | 0.0-11.2, 1.00 (reference); 11.3-22.0, 0.97 (0.66,1.42); 22.1-43.4, 1.28 (0.87,1.87); 43.5-273.0, 1.48 (0.98,2.23) | 7 | First-degree family history of breast cancer, number of full-term pregnancies, height, change in BMI between age 20 and 30 years, total months of breastfeeding, mean daily alcohol consumption and the other types of activity |
| John, 2003 | America | | Female | Population-based | | 2798,  1250 | Lifetime | Breast cancer | | Hours/week | Premenopausal: <3.3,1.0 (reference); 3.3-7.3, 0.88 (0.62-1.24); ≥7.4, 0.81 (0.53-1.24) Postmenopausal: <3.4,1.0 (reference); 3.4-7.8, 0.88 (0.70-1.12); ≥7.9, 1.20 (0.90-1.61) | 7 | Age, race/ethnicity, country of birth, education, family history of breast cancer, prior biopsy for benign breast disease, age at menarche, parity, age at first full-term pregnancy, breast-feeding, BMI, and other types of activity |
| Zhang, 2003 | Asia | | Female | Population-based | | 3067,  1494 | Past 10 years | Breast cancer | | Hours/day | <1, 1 (reference); 1-<2, NA; 2-<3. 1.08 (0.85-1.36); 3-<4, 1.10 (0.87-1.39); ≥4, 0.88 (0.68-1.13) | 7 | Age, education, level personal income of last year, age at menarche, menopause, prenancy, the number of live birth, breastfeeding, oral contraceptive, hormone medicine, body index, benign breast disease, alcohol, tea, vitamin/calcium, occupation, family history of breast cancer and fresh beans |
| Friendenreich, 2004 | America | | Male | Population-based | | 2051,  988 | Lifetime | Prostate cancer | | MET-hours/week | 0-<7.79, 1.0 (reference); ≥7.79-<16.0, 1.12 (0.86-1.46); ≥16.0-<26.8, 1.13 (0.87-1.47); ≥26.8, 1.36 (1.05-1.76) | 7 | Age, region, education, BMI, waist/hip ratio, total caloric intake, average lifetime total alcohol intake, first degree family history of prostate cancer, number of times had prostate-specific antigen test done, number of digital rectal examinations, and other types of activity |
| Matthews, 2005 | Asia | | Female | Population-based | | 1677,  832 | Past 10 years | Endometrial cancer | | Hours/day | 0-2, 1.00 (reference); 3, 0.81 (0.62-1.05); 4, 0.69 (0.51-0.92); >5, 0.62 (0.46-0.85) | 6 | Age, age at menarche, menopausal status and age, number of pregnancies, oral contraceptive use, current smoking, ever drinking, family history of cancer, education, height, and BMI |
| Cerhan, 2005 | America | | Both | Population-based | | 866,  457 | Past 1 year | Non-Hodgkin lymphoma | | Yes/no | No, 1 (reference); yes, 1.23 (0.92-1.63) | 6 | Age, gender, race, and study center |
| Kruk, 2007 | Europe | | Female | Hospital-based | | 551,  250 | Lifetime | Breast cancer | | MET-hours/week, hours/week | MET-hours/wk: <38, 1.00 (reference); 38-60, 0.51 (0.29-0.87); >60, 0.54 (0.31-0.94) Hours/wk: <13, 1.00 (reference); 13-20, 0.49 (0.28-0.86); >20, 0.32 (0.18-0.58) | 7 | Age, BMI, marital conditions, parity, active cigarette smoking |
| Wiklund, 2008 | Europe | | Male | Population-based | | 2082,  1,262 | Lifetime | Prostate cancer | | MET-hours/week | 0-<2.1, 1.00 (reference); ≥2.1-<2.9, 1.34 (0.96-1.87);≥2.9-4.6, 1.33 (1.02-1.74); ≥4.6, 1.44 (1.08-1.92) | 7 | Age, region, education level, BMI, alcohol intake, family history of prostate cancer, personal history of diabetes, total energy intake |
| Peplonska, 2008 | Europe | | Female | Population-based | | 4502,  2,176 | Audult lifetime | Breast cancer | | MET-hours/wk | <29.8, 1.00 (reference); 29.8-<46.5, 0.81 (0.67-0.96); 46.5-<69.6, 0.77 (0.64-0.93); ≥69.6, 0.88 (0.73-1.07) | 7 | Age, study site, education, BMI, age at menarche, menopausal status, age at menopause (in postmenopausal women), number of full-term births, age at first full-term birth, breastfeeding, family history of breast cancer, previous screening mammography, and other types of activity |
| Mathew, 2009 | Asia | | Female | Hospital-based | | 3739,  1866 | 1 year before diagnosis/ at the time of data collection | Breast cancer | | Min/day | Premenopausal: <180, 1.00 (reference); 3-239, 0.94 (0.63-1.40); 240-299, 0.80 (0.55-1.17); 300-359, 0.48 (0.32-0.72); ≥360, 0.70 (0.48-1.02) Postmenopausal: <180,1.00 (reference); 180-239, 0.84 (0.59-1.19); 240-299, 0.77 (0.54-1.09); 300-359, 0.49 (0.34-0.72); ≥360, 0.51 (0.35-0.73) | 8 | Age, center, religion, marital status, education, socioeconomic status, residence status, occupation, BMI, waist and hip sizes, parity, age at first childbirth, and duration of breast feeding |
| Rundel, 2010 | Europe | | Both | Population-based | | 878,  230 | Recent years | Lung cancer | | MET-hours/wk | 0-19.00, 1 (reference); >19.00-36.5, 1.07 (0.66-1.71); >36.5-63.30, 1.08 (0.67-1.75); >66.30, 1.55 (0.91-2.63) | 8 | Age, sex, smoking status, country of recruitment, follow-up time and total years of smoking |
| Wen, 2010 | Asia | | Both | Hospital-based | | 900,  300 | 1 year before diagnosis | Gastric cancer | | Times/week | 0, 1.00 (reference); ≤3, 0.95 (0.74-1.67); ≥4, 0.83 (0.57-1.44) | 7 | Age, sex, BMI, family history, smoking, drinking, fresh fruit and fresh vegetables |
| Inumaru, 2012 | America | | Female | Hospital-based | | 279,  93 | At 20 years old | Breast cancer | | Yes/no | No, 1 (reference); yes, 0.72 (0.34-1.55) | 5 | N/A |
| Kobayashi, 2013 | America | | Female | Population-based | | 2282,  1110 | Lifetime | Breast cancer | | MET-hours/week | Premenopausal: 0, 1.00 (reference); ≤13.1, 0.97 (0.64-1.48); 13.11-54.8, 0.92 (0.60-1.39); >54.8, 0.94 (0.62-1.43) Postmenopausal: 0, 1.00 (reference); ≤21.5, 0.78 (0.58-1.06); 21.51-61.1, 0.70 (0.52-0.95); >61.1, 0.58 (0.43-0.79) | 5 | Age, study center, education, ethnicity, ever oral contraceptive use, number of live births(only for Pre-menopausal) and other domains of moderate-to-vigorous physical activity |
| Brerner, 2014 | Europe | | Both | Population-based | | 1089,  566 | Lifetime | Pancreatic cancer | | MET-hours/week | Men: 0,1 (reference); ≤15.7, 0.71 (0.40-1.25); >15.70-≤34.2, 1.06 (0.58-1.96); >34.2, 0.51 (0.23-1.12) Women: 0, 1 (reference);≤15.7, 1.06 (0.39-2.87); >15.70-≤34.2, 0.70 (0.35-1.38); >34.2, 0.84 (0.46-1.53) | 6 | Age, center , education level, alcohol intake, tobacco smoking, BMI, history of diabetes, hypertension, gallstones, and pancreatitis |
| Hou, 2014 | Africa | | Female | Hospital-based & population-based | | 1022,  337 | Lifetime | Breast cancer | | MET-hours/day | <0.4, 1 (reference); 0.5-1.9, 0.38 (0.26-0.57); 2.0-7.9, 0.38 (0.26-0.56); >7.9, 0.25 (0.17-0.37) | 6 | Age, education, BMI, and study site |
